# Salidroside Ameliorated Intermittent Hypoxia-Aggravated Endothelial Barrier Disruption and Atherosclerosis *via* the cAMP/PKA/RhoA Signaling Pathway

**DOI:** 10.3389/fphar.2021.723922

**Published:** 2021-08-24

**Authors:** Linyi Li, Yunyun Yang, Huina Zhang, Yunhui Du, Xiaolu Jiao, Huahui Yu, Yu Wang, Qianwen Lv, Fan Li, Qiuju Sun, Yanwen Qin

**Affiliations:** ^1^ The Key Laboratory of Upper Airway Dysfunction-Related Cardiovascular Diseases, Beijing Anzhen Hospital, Beijing Institute of Heart, Lung and Blood Vessel Diseases, Capital Medical University, Beijing, China; ^2^ The Key Laboratory of Remodeling-Related Cardiovascular Diseases, Beijing Anzhen Hospital, Ministry of Education, Capital Medical University, Beijing, China; ^3^ Beijing Institute of Heart, Lung and Blood Vessel Disease, Beijing, China

**Keywords:** atherosclerosis, obstructive sleep apnea-hypopnea syndrome, intermittent hypoxia, salidroside, endothelial barrier

## Abstract

**Background:** Endothelial barrier dysfunction plays a key role in atherosclerosis progression. The primary pathology of obstructive sleep apnea-hypopnea syndrome is chronic intermittent hypoxia (IH), which induces reactive oxygen species (ROS) overproduction, endothelial barrier injury, and atherosclerosis. Salidroside, a typical pharmacological constituent of Rhodiola genus, has documented antioxidative, and cardiovascular protective effects. However, whether salidroside can improve IH-aggravated endothelial barrier dysfunction and atherosclerosis has not been elucidated.

**Methods and results:** In normal chow diet-fed ApoE^−/−^ mice, salidroside (100 mg/kg/d, p. o.) significantly ameliorated the formation of atherosclerotic lesions and barrier injury aggravated by 7-weeks IH (21%–5%–21%, 120 s/cycle). In human umbilical vein endothelial cells (HUVECs), exposure to IH (21%–5%–21%, 40 min/cycle, 72 cycles) decreased transendothelial electrical resistance and protein expression of vascular endothelial cadherin (VE-cadherin) and zonula occludens 1. In addition, IH promoted ROS production and activated ras homolog gene family member A (RhoA)/Rho-associated protein kinase (ROCK) pathway. All of these effects of IH were reversed by salidroside. Similar to salidroside, ROCK-selective inhibitors Y26732, and Fasudil protected HUVECs from IH-induced ROS overproduction and endothelial barrier disruption. Furthermore, salidroside increased intracellular cAMP levels, while the PKA-selective inhibitor H-89 attenuated the effects of salidroside on IH-induced RhoA/ROCK suppression, ROS scavenging, and barrier protection.

**Conclusion:** Our findings demonstrate that salidroside effectively ameliorated IH-aggravated endothelial barrier injury and atherosclerosis, largely through the cAMP/PKA/RhoA signaling pathway.

## Introduction

Atherosclerotic cardio- or cerebrovascular diseases are common causes of morbidity and mortality worldwide (Gibbons et al., 2021). Atherosclerosis is the pathological basis of peripheral arterial disease, coronary heart disease, and stroke. In addition to traditional risk factors such as hereditary susceptibility, aging, hypertension, obesity, diabetes, and smoking, obstructive sleep apnea-hypopnea syndrome (OSA) that affect up to 100 million people worldwide has emerged as a new significant and independent risk factor for atherosclerosis (Ali et al., 2014; Souza et al., 2021). OSA is characterized by repetitive episodes of hypopnea or apnea due to upper airway collapse during sleep (Lévy et al., 2015). OSA prevalence in patients with coronary heart disease ranges from 30 to 58% (Bradley and Floras, 2009). OSA causes endothelial dysfunction (Hoyos et al., 2015; Khalyfa et al., 2016), unstable plaque characteristics (Trzepizur et al., 2014; Konishi et al., 2019), and atherosclerosis (Wattanakit et al., 2008; Ma et al., 2016; Zhou et al., 2017). Recently, one of the largest studies in this area to date (*n* = 2009) found that OSA is independently associated with increased carotid intima-media thickness in a dose-responsive manner (Souza et al., 2021), reinforcing the role of OSA as a potential nontraditional risk factor for atherosclerosis.

Chronic intermittent hypoxia (CIH), a main feature of OSA, is characterized by repeated circulating hypoxemia and reoxidation (Lévy et al., 2015). CIH induces atherosclerosis in murine models and plays a critical role in OSA-associated cardiovascular morbidities (Drager et al., 2013; Gautier-Veyret et al., 2013; Gileles-Hillel et al., 2014). Endothelial barriers are crucial for maintaining vascular homeostasis. In atherosclerosis, endothelial barrier function is impaired, which triggers the formation of atherosclerotic plaques (Sun et al., 2011; Döring et al., 2017; Pi et al., 2018). CIH has been shown to increase vascular permeability and endothelial barrier dysfunction (Makarenko et al., 2014). However, whether CIH destroys vascular endothelial barriers to promote atherosclerosis remains unknown.

CIH can reportedly increase reactive oxygen species (ROS) generation, which subsequently cause vascular endothelial cadherin (VE-cadherin) cleavage, endothelial barrier injury, and dysfunction (Makarenko et al., 2014; Harki et al., 2021). Ras homolog gene family member A (RhoA) and its downstream Rho-associated protein kinase (ROCK) play crucial roles in both oxidative stress and endothelial barrier function (Shimokawa and Satoh, 2015; Komarova et al., 2017; Dee et al., 2019; Cong and Kong, 2020). cAMP/protein kinase A (PKA) signaling is a potent protective pathway to stabilize the endothelial barrier in response to oxidative stress, and an essential negative regulator of RhoA/ROCK (Qiao et al., 2003; Cong and Kong, 2020).

Salidroside is a typical pharmacological constituent found in medicinal plants under the Rhodiola genus. In addition to their anti-fatigue and anti-hypoxia roles in traditional medicine, Rhodiola extract, and salidroside also displayed medicinal properties as anti-cardiovascular diseases agents, which was mainly attributed to their antioxidant effects (Li et al., 2017; Zhao et al., 2021). *Rhodiola crenulata* exerts protective effects on hypoxia/high glucose-induced endothelial dysfunction and CIH-induced cardiac apoptosis (Lai et al., 2015; Chang et al., 2018; Huang et al., 2020). *Rhodiola rosea* extract attenuated pulmonary hypertension in chronic hypoxic rats (Kosanovic et al., 2013) and inhibited atherosclerosis formation in high fat diet-fed rabbits (Shen et al., 2008). Salidroside exhibited activity similar to that of Rhodiola extract. Salidroside improved CIH-mediated myocardial cell apoptosis (Zhong et al., 2010; Lai et al., 2014), chronic persistent hypoxia-induced pulmonary hypertension (Chen et al., 2016) and high fat diet-related atherosclerosis (Xing et al., 2015; Bai et al., 2020; Song et al., 2021) in animal models. Moreover, salidroside suppresses oxidative stress-induced HUVECs cell injury (Zhao et al., 2013; Zhu et al., 2016). Our previous study also demonstrated that salidroside could improve homocysteine-induced endothelium-dependent relaxation dysfunction by reducing oxidative stress (Leung et al., 2013). However, the effect of salidroside on endothelial barrier and CIH-aggravated atherosclerosis has not been reported. Wang et al. found that salidroside attenuated inflammatory responses by inhibiting the RhoA/ROCK pathway in vascular smooth muscle cells (Wang et al., 2021). Guan et al. reported that salidroside attenuated hydrogen peroxide-induced cell damage by elevating cAMP levels (Guan et al., 2011). Therefore, in the present study, we hypothesized that salidroside protects the integrity of endothelial barriers and alleviates CIH-aggravated atherosclerosis through effects on cAMP/PKA/RhoA signaling.

## Materials and Methods

### Reagents

The following drugs were used: salidroside (98% purity; Sigma-Aldrich, St. Louis, MO, United States), H-89 dihydrochloride hydrate (PKA-specific inhibitor; Sigma-Aldrich), Y-27632 dihydrochloride (ROCK-specific inhibitor; Tocris Bioscience, Bristol, United Kingdom), and Fasudil hydrochloride (ROCK-specific inhibitor, Tocris Bioscience). All drugs were freshly dissolved in medium at the beginning of each experiment. The following primary antibodies were purchased from the cited commercial sources: zonula occludens 1 (ZO-1, Invitrogen, Carlsbad, CA, United States), VE-cadherin (Santa Cruz Biotechnology, Santa Cruz, CA, United States), CD31 (Invitrogen), ROCK (Abcam, Cambridge, United Kingdom), RhoA (Invitrogen and Abcam), and GAPDH (Abcam). FITC-conjugated anti-rabbit and anti-mouse secondary antibodies were purchased from Abcam.

### Experiments in ApoE^-/-^ Mice

All animal handling complied with the standard animal welfare regulations of Capital Medical University (Beijing, China). The Animal Subjects Committee of Capital Medical University approved the animal study protocol. Animals were randomly assigned to the experimental groups. Ten-week-old male ApoE^-/-^ mice were purchased from HFK Bioscience (Beijing, China). All animals were fed a normal chow diet and maintained in a controlled environment with 12-h light/dark cycles, temperature of 22 ± 2°C, and humidity of 50 ± 2%. To deliver CIH, mice were housed in an OxyCycler A84 System (BioSpherix, Redfield, NY, United States). Briefly, a gas control system regulated the room airflow (N_2_ and O_2_). A series of programs and flow regulators enabled manipulation of the fraction of inspired O_2_ from 21 to 5.0% over a 90-s period, followed by rapid reoxygenation to normal air levels via a burst of 100% O_2_ in the succeeding 30-s period. Hypoxic events occurred at a rate of one event per 120 s throughout the 12-h light period. During the 12-h dark period, CIH animals were maintained in a normoxic environment. Non-CIH mice were exposed to normoxia at all times. Mice were randomly assigned to one of three groups (*n* = 8 in each group): 1) control, mice not subjected to CIH, N.S., p. o.; 2) CIH, mice subjected to CIH, N.S., p. o.; and 3) CIH plus salidroside (CIH + Sali), mice subjected to CIH and salidroside (100 mg/kg/d), p. o. After 7 weeks, all mice were anesthetized with pentobarbital sodium and arterial blood were collected by cardiac puncture from the left ventricle. Then mice were perfused with ice-cold saline solution and their ascending aortas were collected for Oil Red O staining. Plaque sizes were quantified using Image-Pro Plus software (Media Cybernetic, Tokyo, Japan). Serial cross-sections (8 μm) of the heart throughout the entire aortic valve area were cut with a cryostat (CM 1900; Leica, Wetzlar, Germany). Sections stained with VE-cadherin (1:50; Cat. No. Sc-9989, Santa Cruz), CD31 (1:50; Cat. No. PA5-16301, Invitrogen), ROCK (1:100; Cat. No. 39749, Abcam), or RhoA (1:50; Cat. No. PA5-87403, Invitrogen) were digitally scanned (Pannoramic DESK, P-MIDI, P250; 3DHISTECH, Budapest, Hungary) to obtain images for analysis by a previously described method (Yang et al., 2020). The methods for oral glucose tolerance test, insulin tolerance test, and biochemical analysis are included in the Supplementary Material.

### Cell Culture and Treatment

All experiments using human umbilical vein endothelial cells (HUVECs) were approved by the Medical Ethics Committee of Beijing Anzhen Hospital (Approval No. 2017005) and conducted in accordance with guidelines of the Declaration of Helsinki. Written informed consent was obtained from all participating donors. Human umbilical cord veins were donated by the Maternal and Child Care Service Centre in Beijing, China. HUVECs were isolated, purified, and identified as described previously (Qin et al., 2012). Cells were cultured in VascuLife Basal Medium (Lifeline Cell Technology, Frederick, MD) supplemented with 2% fetal bovine serum, 5 ng/ml recombinant human epidermal growth factor, 10 mM l-glutamine, 1 mg/ml hydrocortisone hemisuccinate, 0.75 U/mL heparin sulfate, 0.2% EnGS (Lifeline Cell Technology), 10^4^ U/mL penicillin, and 10^4^ U/mL streptomycin (HyClone, Logan, UT) in humidified incubator with 95% air (v/v) and 5% CO_2_ (v/v) at 37°C. HUVECs were used from passages 2–5.

The intermittent hypoxia (IH) protocol consisted of alternating cycles of 20-min hypoxia (1% O_2_ and 5% CO_2_) and 20-min reoxygenation (21% O_2_ and 5% CO_2_), using a BioSpherix OxyCycler C42 system (BioSpherix, Redfield, NY, United States). The total duration of one cycle was 40 min. Cultured HUVECs were pretreated with the indicated concentration of drugs for 2 h and then exposed to the indicated number of cycles of IH.

### Measurement of Transendothelial Electrical Resistance

HUVECs were seeded in the top chambers of 24-well Transwell plates (8.0-µm pore size; Cat. No. 35-3097; Becton-Dickinson, Franklin Lakes, NJ, United States) at a density of 1 × 10^4^ cells/well. Volumes of culture media in the top and lower chambers were 300 and 700 μL, respectively. TEER was measured using a Millicell-ERS (MERS00002; Millipore, Burlington, MA, United States), as previously described (Li et al., 2015). Values for blank wells and cell wells represented the blank resistance (R blank) and sample resistance (R Sample), respectively. The following calculation was performed: TEER = (R Sample)–(R blank). The effect of IH on TEER was determined in HUVECs after 9, 18, 36, 72, and 108 cycles of IH exposure. The effect of drugs on permeability was determined in HUVECs pretreated with salidroside (10 µM or 100 µM), H-89 (10 μM), Y27632 (10 µM), or Fasudil (10 µM) for 2 h before exposure to either normoxia or 72 cycles of IH.

### Measurement of HUVEC Permeability to FITC-Dextran

HUVECs were seeded in the top chambers of 24-well Transwell plates (0.4-µm pore size polyester membrane inserts; Corning, Corning, NY, United States) at a density of 1 × 10^4^ cells/well. Volumes of culture media in the top and lower chambers were 200 and 700 μL, respectively. Permeability was measured by adding 1 mg/ml FITC-dextran (70 kDa; Sigma-Aldrich) immediately after IH exposure. Following a 20-min incubation with FITC-dextran, 100 µL of medium was aspirated from each of the lower chambers into wells of black 96-well microplates (Nunc, Roskilde, Denmark). The fluorescence value of each well was determined by an EnSpire 2300 Multimode Plate Reader (PerkinElmer, Waltham, MA, United States) at a 490-nm excitation wavelength and 525-nm emission wavelength. The effect of IH on permeability of HUVECs to FITC-dextran was determined after 9, 18, 36, 72, and 108 cycles of IH exposure. The effect of salidroside on permeability was determined in HUVECs pretreated with salidroside (10 µM or 100 µM) for 2 h before exposure to either normoxia or 72 cycles of IH.

### Assay of ROS Production

Intracellular ROS was detected by fluorescence microscopy and quantitated by a plate reader using 2ʹ,7ʹ-dichlorofluorescein diacetate (DCFH-DA, Sigma-Aldrich) as the probe, as previously described (Naha et al., 2010). HUVECs were cultured in black 96-well microplates (Nunc) at a density of 4 × 10^5^ cells/mL in 100 µL of medium. After exposure to drugs under normoxia or IH, microplates were washed with 100 µL/well of phosphate-buffered saline (PBS) and then 100 µL of 10 µM DCFH-DA was added to each well. After incubating the microplates at 37°C for 20 min and washing three times with PBS, an EnSpire 2300 plate reader was used to quantify the fluorescence of each well at a 490-nm excitation wavelength and 525-nm emission wavelength. Fluorescence images were obtained with a Ni-U Nikon Upright Microscope equipped with a DS-Ri2 color charge-coupled device (Nikon, Tokyo, Japan). The effect of each drug on ROS production was determined in HUVECs pretreated with salidroside (10 µM or 100 µM), Tiron (10 µM), H-89 (10 µM), Y27632 (10 µM), or Fasudil (10 µM) for 2 h before exposure to either normoxia or 72 cycles of IH.

### GST Pull-Down Assay to Evaluate RhoA Activity

After experimental treatments, HUVECs were lysed and cell lysates were collected. The supernatant was centrifuged at 4°C at 700 g for 10 min. The resulting protein was quantified by bicinchoninic acid method, and GST-RBD fusion was added to the total protein samples of each group. After incubation at 4°C for 1 h, protein samples were centrifuged at 1900 g for 3 min. The precipitated protein was GST-bound. Expression of RhoA protein in total and GST-bound samples was detected by western blot using a rabbit monoclonal RhoA antibody (1:1,000; Cat. No. ab187027, Abcam). RhoA protein in the total protein sample was expressed as total RhoA, while RhoA protein in the GST-bound protein sample was expressed as GTP-RhoA. The ratio of GTP-RhoA expression to total RhoA expression reflected the activity level of RhoA in HUVECs.

### Measurement of Intracellular cAMP

HUVECs were cultured by seeding 100 µL of cell suspension with a density of 1 × 10^5^ cells/mL in wells of 24-well plates. After exposure to drugs under normoxia or IH, intracellular cAMP was extracted with hydrochloric acid (HCl, 0.1 M). cAMP concentrations were assessed with a cAMP Assay Kit (Cat. No. ab65355, Abcam) according to the manufacturer’s protocol. The effect of drugs on intracellular cAMP was determined in HUVECs pretreated with salidroside (10 µM or 100 µM) for 2 h before exposure to either normoxia or 72 cycles of IH.

### Immunostaining for HUVECs

HUVECs were exposed to either normoxia or 72 cycles of IH, and then fixed with methanol for 30 min at −40°C. After blocking with 3% bovine serum albumin for 30 min, cells were incubated overnight with primary antibodies against VE-cadherin (1:100; Cat. No. 36-1900, Invitrogen) and ZO-1 (1:100; Cat. No. 33-9100, Invitrogen). The following day, cells were washed three times with PBS and incubated with FITC-conjugated anti-mouse and TRITC-conjugated anti-rabbit secondary antibodies (1:200) for 1 h at room temperature. Images of immunofluorescence staining were acquired with a Leica TCS SP2 confocal laser-scanning microscope.

### Western Blot Analysis

To extract protein from HUVECs, a protein extraction kit containing protease inhibitors and a protein phosphatase inhibitor cocktail was used (Thermo Fisher Scientific). Equal amounts of protein (30 µg/lane) were separated by 10% sodium dodecyl sulfate polyacrylamide gel electrophoresis. Blots were probed overnight at 4°C with primary antibodies (1:1,000), washed with Tris-buffered saline containing Tween 20, and incubated with secondary antibodies (1:10,000; ZSGB-BIO, Beijing, China) for 1 h at room temperature. Finally, blots were washed, incubated with SuperSignal™ WestFemto Maximum Sensitivity Substrate (Thermo Fisher Scientific), and analyzed using a ChemiDoc™ Touch Imaging System (Bio-Rad, Hercules, CA, United States).

### Statistical Analysis

Data were analyzed using Prism 5.0 software (GraphPad Software, San Diego, CA) and are presented as mean ± standard deviation (SD). In all cases, the results of at least three independent experiments were used. Statistical comparisons between two groups were performed using Student’s *t*-test. Multiple comparison tests used one-way analysis of variance with Bonferroni’s procedure. Values of *p* < 0.05 were considered statistically significant.

## Results

### Salidroside Alleviated Atherosclerotic Lesion Formation in CIH-Exposed ApoE^-/-^ Mice

**FIGURE 1:**
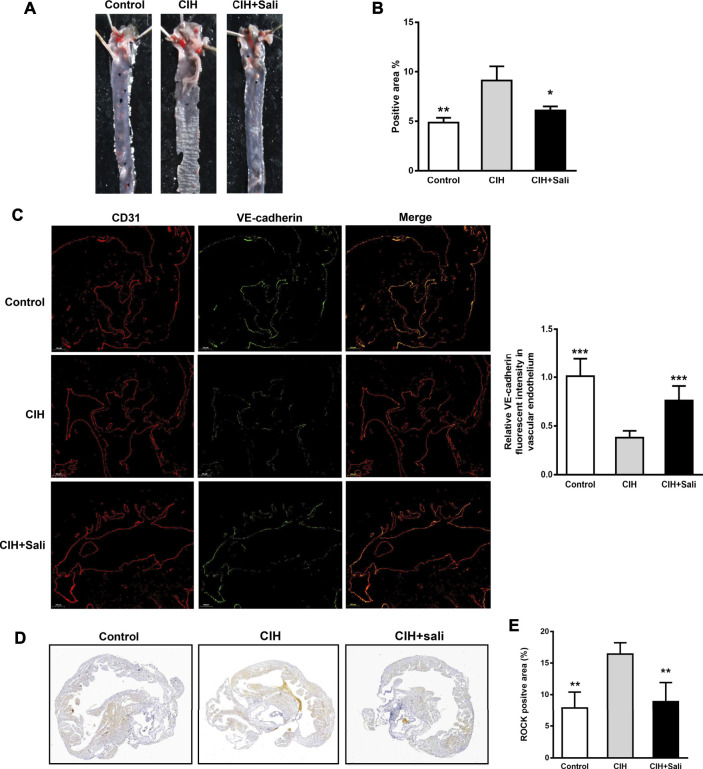
Salidroside attenuated CIH-aggravated atherosclerosis in ApoE^-/-^ mice. Male ApoE^-/-^ mice were randomly divided into normoxia (control), CIH, and CIH + Sali groups. After experimental intervention for 7 weeks, mice were sacrificed under anesthesia **(A)** Representative images of aortic Oil Red O staining **(B)** Quantitative summary of atherosclerotic lesions in aortas (*n* = 4) **(C)** Immunofluorescence staining of VE-cadherin and CD31 and relative fluorescent intensity of VE-cadherin in the endothelium of aortic roots (5 × magnification, *n* = 3 sections per tissue, at least three analysis sites per slide) **(D)** Plaques of aortic roots were stained with ROCK (2 × magnification) **(E)** Quantitative summary of ROCK expression in aortic root plaques (*n* = 4). CIH, chronic intermittent hypoxia; Sali, salidroside; VE-cadherin, vascular endothelial cadherin; ROCK, Rho-associated protein kinase. All data are presented as mean ± SD. *p < 0.05, **p < 0.01, and ***p < 0.001 compared with CIH group.

Analysis of Oil Red O staining revealed a significant increasein lipid accumulation (*p* < 0.01) in aortic sinus tissue of CIH mice compared with control mice, as well as a significant decrease (*p* < 0.05) in CIH + Sali mice compared with CIH mice (Figures 1A,B). Salidroside did not alter fasting blood glucose, oral glucose tolerance, fasting blood triglyceride, fasting total blood cholesterol, or insulin sensitivity levels in CIH-exposed ApoE^-/-^ mice (Supplementary Figures S1A–E). To determine the effect of salidroside on endothelial barriers in CIH-exposed ApoE^-/-^ mice, immunohistochemistry, and immunofluorescence double staining was conducted to measure expression levels of VE-cadherin, CD31, and ROCK in aortic plaques. As shown in Figures 1A,C–E, a significant decrease in VE-cadherin (*p* < 0.001) and significant increase of ROCK (*p* < 0.01) were observed in the CIH group compared with the control group. Meanwhile, salidroside significantly increased VE-cadherin (*p* < 0.001) and significantly attenuated ROCK expression (*p* < 0.01).

### Salidroside Ameliorated IH-Induced Endothelial Barrier Dysfunction in HUVECs

**FIGURE 2:**
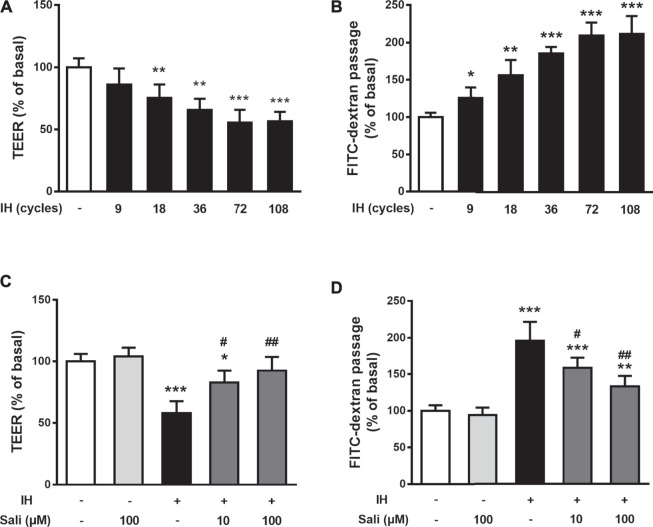
Salidroside ameliorated IH-induced endothelial barrier dysfunction in HUVECs. The effects of IH on TEER **(A)** and permeability to FITC-dextran **(B)** were determined in HUVECs after 9, 18, 36, 72, 108 cycles of IH exposure. Effects of Sali on TEER **(C)** and permeability to FITC-dextran **(D)** were determined in HUVECs pretreated with Sali (10 µM or 100 µM) for 2 h before exposure to either normoxia or 72 cycles of IH. IH, intermittent hypoxia [one cycle includes reducing O_2_ for a 20-min hypoxia period (5% O_2_ and 5% CO_2_) followed by 20-min reoxygenation (21% O_2_ and 5% CO_2_)]; Sali, salidroside; TEER, transendothelial electrical resistance. All data are presented as mean ± SD. #*p* < 0.05 and ##*p* < 0.01 compared with IH group; *p < 0.05, **p < 0.01, and ***p < 0.001 compared with normoxia group.

Endothelial barrier function was assayed by measuring TEER and permeability to FITC-dextran in HUVECs immediately after IH exposure. Compared with the normoxia group, IH exposure significantly decreased TEER (*p* < 0.001) and increased permeability to FITC-dextran (*p* < 0.001) of HUVECs in a time-dependent manner, reaching a plateau with 72 cycles of IH (Figures 2A,B). Therefore, the following studies were performed on HUVECs exposed to 72 cycles of IH. Pretreatment of HUVECs with salidroside (10 µM or100 µM) dose-dependently and significantly increased TEER (*p* < 0.01) and decreased permeability to FITC-dextran (*p* < 0.01) compared with the IH group (Figures 2C,D).

### Salidroside Ameliorated the Suppressive Effect of IH on VE-Cadherin and ZO-1 Expression in HUVECs

**FIGURE 3:**
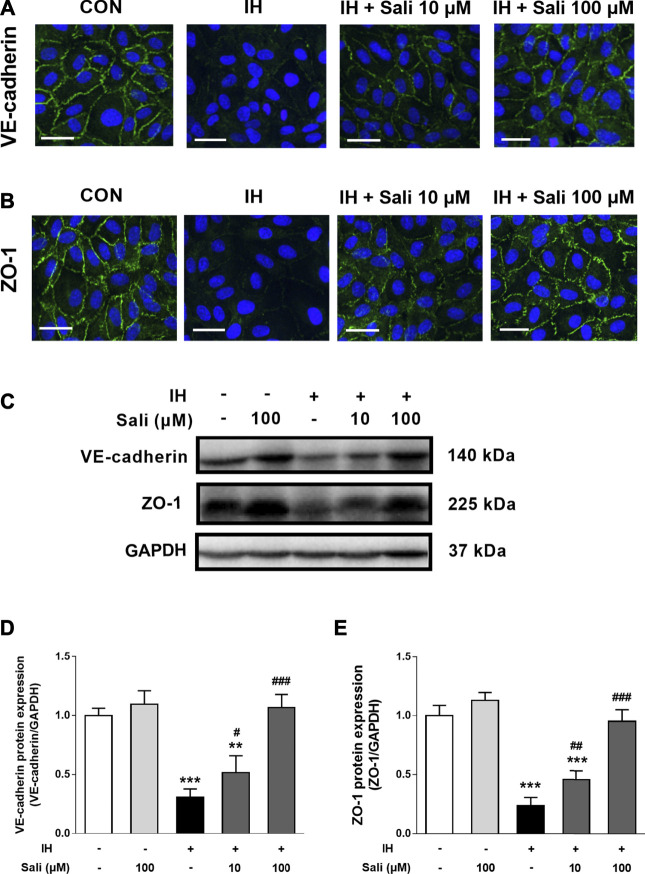
Salidroside ameliorated the suppressive effect of IH on VE-cadherin and ZO-1 expression in HUVECs. HUVECs were pretreated with Sali (10 µM or 100 µM) for 2 h before exposure to 72 cycles of IH **(A)** Immunofluorographs of VE-cadherin (20 × magnification; Bar = 50 µm) and **(B)** ZO-1 (20 × magnification; Bar = 50 μm) **(C)** VE-cadherin and ZO-1 protein levels were measured by western blot analysis **(D–E)** Relative protein levels of VE-cadherin and ZO-1 were quantified by densitometry analysis. IH, intermittent hypoxia; Sali, salidroside; VE-cadherin, vascular endothelial cadherin; ZO-1, zonula occludens 1. All data are presented as mean ± SD. #*p* < 0.05, ##*p* < 0.01, and ###*p* < 0.001 compared with IH group; **p < 0.01 and ***p < 0.001 compared with normoxia group.

As shown in Figures 3A,B, IH-exposed HUVECs displayed obvious disruptions of VE-cadherin and ZO-1 by immunofluorescence staining that were dose-dependently ameliorated by salidroside pretreatment. Similarly, western blot assay results showed that IH exposure significantly reduced protein expression levels of VE-cadherin (*p* < 0.001) and ZO-1 (*p* < 0.001), but this effect was largely reversed by salidroside pretreatment (*p* < 0.001, Figures 3C–E).

### Salidroside Attenuated IH-Induced ROS Overproduction in HUVECs

**FIGURE 4:**
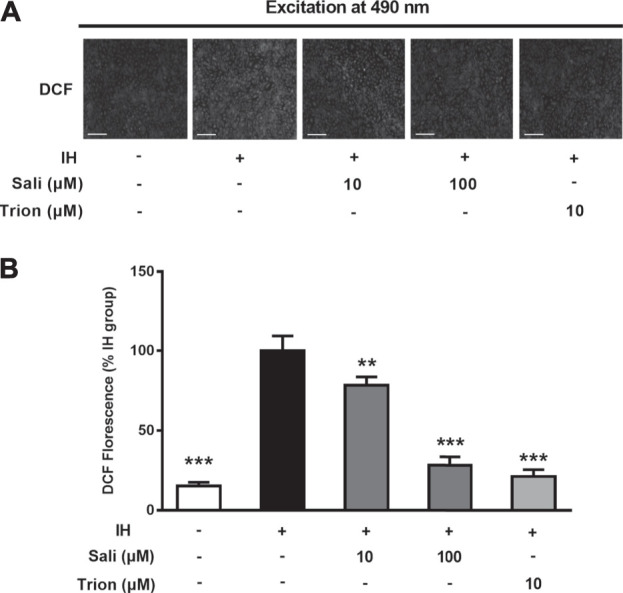
Salidroside attenuated IH-induced ROS overproduction in HUVECs. Intracellular ROS levels were determined in HUVECs pretreated with Sali (10 µM or 100 µM) or Tiron (10 µM) for 2 h before exposure to either normoxia or 72 cycles of IH. DCFH-DA was used as a fluorescent probe for ROS. Tiron was used as a positive control **(A)** Fluorographs of intracellular ROS were acquired with a fluorescence microscope (5 × magnification; Bar = 500 µm) **(B)** Fluorescence quantification results of intracellular ROS. IH, intermittent hypoxia; Sali, salidroside; DCFH-DA, 2ʹ,7ʹ-dichlorofluorescein diacetate; ROS, reactive oxygen species. All data are presented as mean ± SD. *p < 0.05 and ***p < 0.001 compared with IH group.

ROS play a major role in IH-related endothelial dysfunction. To determine whether salidroside attenuated IH-induced oxidative stress in HUVECs, intracellular ROS production was measured by a fluorescent method. Immunostaining revealed elevated ROS production in the IH group, which was decreased in groups treated with salidroside (10 and 100 μM) or the ROS scavenger Tiron (Figure 4A). Quantification of fluorescence assay results further showed that exposure to 72 cycles of IH significantly increased ROS production (*p* < 0.001) in HUVECs, while salidroside could dose-dependently and significantly suppress ROS production (*p* < 0.001) in IH-exposed HUVECs (Figure 4B).

### Salidroside Elevated Intracellular cAMP in IH-Exposed HUVECs

**FIGURE 5:**
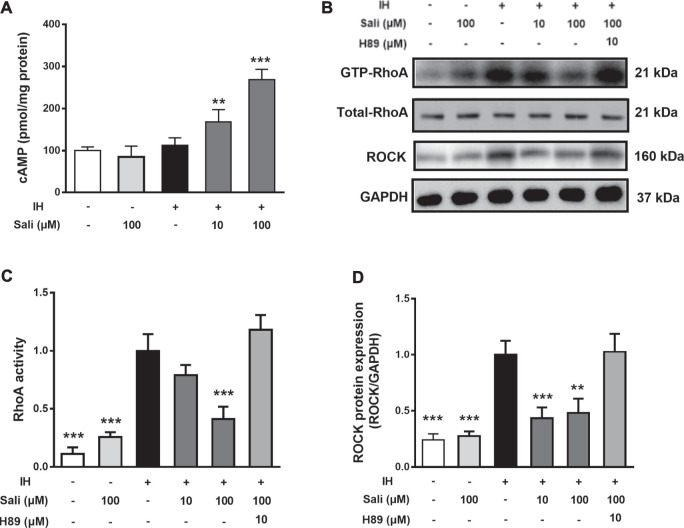
Salidroside suppressed IH-induced elevation of RhoA activity and ROCK protein expression in HUVECs through the cAMP/PKA pathway. HUVECs were pretreated with Sali (10 µM or 100 µM) or the PKA inhibitor H-89 for 2 h before exposure to 72 cycles of IH **(A)** Intracellular cAMP levels, as well as **(B)** RhoA activity and ROCK protein levels were measured **(C–D)** Relative levels of RhoA activity and ROCK protein expression were quantified by densitometry analysis. IH, intermittent hypoxia; Sali, salidroside; RhoA, ras homolog gene family member A; ROCK, Rho-associated protein kinase. All data are presented as mean ± SD. *p < 0.05 and ***p < 0.001 compared with IH group.

Endothelial cAMP is one of the most potent endothelial barrier stabilizers. As shown in Figure 5A, IH exposure did not affect cellular cAMP levels in HUVECs. However, salidroside significantly increased cAMP levels up to 2.4-fold compared with the IH group (*p* < 0.001).

### Salidroside Suppressed IH-Induced Elevation of RhoA Activity and ROCK Protein Expression in HUVECs Through the PKA Pathway

We next examined the effect of salidroside on RhoA activity and ROCK protein expression in HUVECs. Exposure of HUVECs to 72 cycles of IH significantly upregulated RhoA activity (*p* < 0.001) and ROCK (*p* < 0.001) expression. Pretreatment of HUVECs with salidroside significantly reduced IH-induced increases in RhoA activity (*p* < 0.001) and ROCK expression (*p* < 0.01). The PKA-specific inhibitor H-89 significantly attenuated the effect of salidroside on reducing RhoA/ROCK signaling (*p* < 0.001, Figures 5B–D).

### Salidroside Ameliorated IH-Induced ROS Overproduction and Endothelial Barrier Dysfunction in HUVECs *via* cAMP/PKA/RhoA Signaling

**FIGURE 6:**
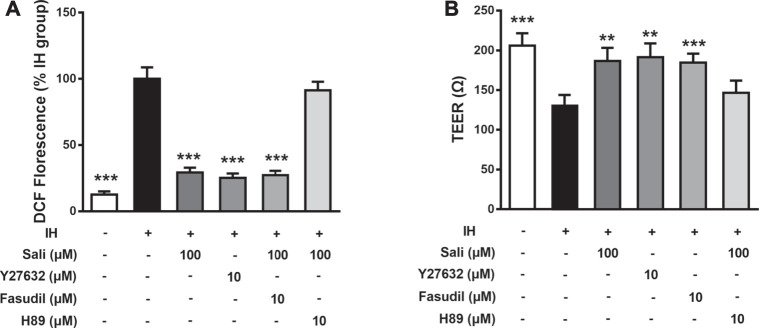
Salidroside ameliorated IH-induced ROS overproduction and barrier dysfunction in HUVECs *via* the cAMP/PKA/RhoA signaling pathway. Effects of drugs on ROS generation **(A)** and TEER **(B)** were determined in HUVECs pretreated with Sali (100 µM), H-89 (10 µM), Y27632 (10 µM), or Fasudil (10 µM) for 2 h before exposure to either normoxia or 72 cycles of IH. IH, intermittent hypoxia; Sali, salidroside; ROS, reactive oxygen species; TEER, transendothelial electrical resistance. All data are presented as mean ± SD of three experiments. ***p < 0.001 compared with IH group.

The ROCK-selective inhibitors Y27632 and Fasudil were employed to further explore the mechanism of salidroside. IH exposure increased ROS production and induced barrier dysfunction in HUVECs, the effects of which were significantly attenuated by salidroside (*p* < 0.01). Similar to salidroside, pretreatment of HUVECs with Y27632 or Fasudil markedly ameliorated IH-induced ROS generation (*p* < 0.001) and barrier disruption (*p* < 0.01 or *p* < 0.001). Moreover, the inhibitory effects of salidroside on IH-induced ROS accumulation (*p* < 0.001) and endothelial barrier disruption (*p* < 0.001) were significantly attenuated by H-89 (Figures 6A,B). Taken together, these results suggest that salidroside ameliorated IH-induced ROS production and barrier dysfunction in HUVECs *via* the cAMP/PKA/RhoA signaling pathway.

## Discussion

The observations of this study can be summarized as follows. Firstly, salidroside ameliorated CIH-induced atherosclerosis progression and barrier injury in normal chow diet-fed ApoE^-/-^ mice. Secondly, salidroside inhibited the deleterious effect of IH on barrier function of HUVECs through effects on cAMP/PKA/RhoA signaling. Furthermore, similar to salidroside, the ROCK inhibitors Y27632 and Fasudil protected HUVECs from IH-induced ROS overproduction and endothelial barrier disruption. This research demonstrates for the first time that salidroside has a protective effect on endothelial barrier function *via* cAMP/PKA/RhoA signaling. These results also suggest that RhoA/ROCK signaling plays important roles in IH-induced oxidative stress and disruption of endothelial integrity, representing a novel mechanism by which sleep breathing disorders exacerbate atherosclerosis.

Clinical and basic studies have demonstrated a causal relationship between CIH and atherosclerosis. Continuous positive airway pressure (CPAP) treatment, which is mainly used to correct CIH in OSA patients, can reduce intima-media thickness and femoral artery wave velocity to improve the early signs of atherosclerosis (Drager et al., 2007; Chen et al., 2017). In addition, CIH promoted atherosclerotic plaque formation in both normal and high cholesterol diet-fed ApoE^-/-^ mice (Drager et al., 2013; Gautier-Veyret et al., 2013; Gileles-Hillel et al., 2014) However, CPAP cannot improve the incidence or mortality rate of cardiovascular diseases in OSA patients, and compliance with CPAP treatment was shown to be very low in actual practice (McEvoy et al., 2016; Peker et al., 2016). Therefore, potential drugs for OSA-related atherosclerotic cardiovascular diseases remain to be further investigated. Salidroside can improve atherosclerosis in high fat diet-fed ApoE^-/-^ mice (Bai et al., 2020; Song et al., 2021). However, the effect of salidroside on CIH-related atherosclerosis has not been reported. Consistent with previous studies, our results indicate that CIH can significantly promote the progression of atherosclerosis in normal chow diet-fed ApoE^-/-^ mice. However, we are the first to report that salidroside can significantly reduce CIH-aggravated plaque progression.

Endothelial barrier dysfunction is the key factor in triggering and aggravating the formation of atherosclerosis. Previous studies showed that endothelial cells in the intima of human atherosclerotic plaques are wider and the endothelial barrier is destroyed, leading to infiltration of various inflammatory cells and the formation of lipid-rich foam cells, a key pathological change of atherosclerosis (Pi et al., 2018). The fundamental basis for barrier function is predominantly maintained by adherens junctions and tight junctions (Komarova et al., 2017). The adherent junction protein VE-cadherin and tight junction protein ZO-1 are important factors to maintain and regulate endothelial barrier function. CIH can reportedly increase vascular permeability and impair endothelial barrier function by VE-cadherin cleavage (Makarenko et al., 2014; Harki et al., 2021). Consistent with previous studies, exposure of HUVECs to IH decreased TEER, increased permeability to FITC-dextran, and suppressed expression of junction proteins VE-cadherin and ZO-1. *In vivo*, CIH significantly reduced VE-cadherin expression in the aortas of ApoE^-/-^ mice. We found that salidroside significantly improved IH-induced endothelial barrier injury in both ApoE^-/-^ mice and HUVECs, suggesting that salidroside may alleviate CIH-aggravated atherosclerosis by improving endothelial barrier damage, and endothelial barrier dysfunction may be a mechanism by which IH aggravates atherosclerosis.

Oxidative stress caused by ROS overproduction is a common mechanism of endothelial barrier dysfunction in both OSA and atherosclerotic diseases (Lévy et al., 2015). The RhoA/ROCK pathway plays a crucial role in ROS augmentation and endothelial barrier function (Shimokawa and Satoh, 2015; Komarova et al., 2017; Dee et al., 2019; Cong and Kong, 2020). Accumulating evidence indicates that Rho-kinase inhibitors have beneficial effects for the treatment of cardiovascular diseases (Dai et al., 2018; Lei et al., 2020). Exposure to IH activated RhoA/Rho-kinase in the aorta and mesenteric arteries of animal models, which is required for IH-induced arterial remodeling, and hypertension (de Frutos et al., 2010; Lu et al., 2017; Li et al., 2018). However, it remains unknown whether the RhoA/ROCK pathway is involved in IH-induced endothelial barrier dysfunction and, if so, what mechanisms are involved. In the present study, we observed that IH increased ROCK expression in the aortas of ApoE^-/-^ mice, as well as ROS generation, RhoA activity, and ROCK expression in HUVECs. Furthermore, the ROCK-selective inhibitors Y27632 and Fasudil attenuated IH-induced ROS overproduction and endothelial barrier disruption. These results suggest that the RhoA/ROCK pathway is essential for IH-induced ROS augmentation and endothelial barrier dysfunction. Because ROS also activates the RhoA/ROCK pathway in endothelial cells (Lu et al., 2017; Li et al., 2018), IH-induced ROS overproduction likely activates RhoA/ROCK pathway, thus creating a vicious cycle for ROS augmentation and endothelial barrier breakdown. Salidroside treatment reversed the increase of ROCK expression in aortas of chronic IH-exposed ApoE^-/-^ mice. Moreover, similar to the ROCK-selective inhibitors Y26732 and Fasudil, salidroside reduced RhoA/ROCK activation and protected HUVECs from ROS overproduction and endothelial barrier disruption induced by IH. These results suggest that the molecular mechanism by which salidroside improved IH-mediated endothelial barrier dysfunction and atherosclerosis was be related to the RhoA/ROCK signaling pathway.

An increase in endothelial cAMP is one of the most potent triggers to stabilize the endothelial barrier against almost any barrier-compromising stimulus (Schlegel and Waschke, 2014; Radeva and Waschke, 2018). Furthermore, cAMP/PKA is an essential negative regulator of RhoA/ROCK (Qiao et al., 2003; Cong and Kong, 2020). Our results suggest that salidroside significantly increased the cAMP level of IH-exposed HUVEC cells (2.4-fold), whereas the PKA-specific inhibitor H-89 attenuated the RhoA/ROCK inhibition, ROS clearance, and barrier protection effects of salidroside. These findings indicate that cAMP/PKA activation acts upstream of RhoA/ROCK inhibition to elicit the endothelial barrier-protective effect of salidroside.

Salidroside did not affect the glucolipid metabolism or insulin sensitivity of CIH-exposed ApoE^-/-^ mice. Therefore, the beneficial effects of salidroside were not secondary to the improvement of glucolipid metabolism or insulin resistance. Moreover, the present results indicate that salidroside has only a moderate regulatory effect under pathophysiological conditions. Under normal conditions, barrier-forming junction proteins undergo dynamic regulation of assembly and disassembly. During these processes, proper Rho activity is very important to maintain the endothelial barrier in a healthy state (Terry et al., 2010). The application of salidroside did not inhibit RhoA activity below a physiological level, but instead maintained RhoA activity at a medium activation level in IH-treated HUVECs, which could provide reliable evidence for the safety of salidroside. However, it remains far from clinical application. The study provides a reference for future clinical research.

**FIGURE 7:**
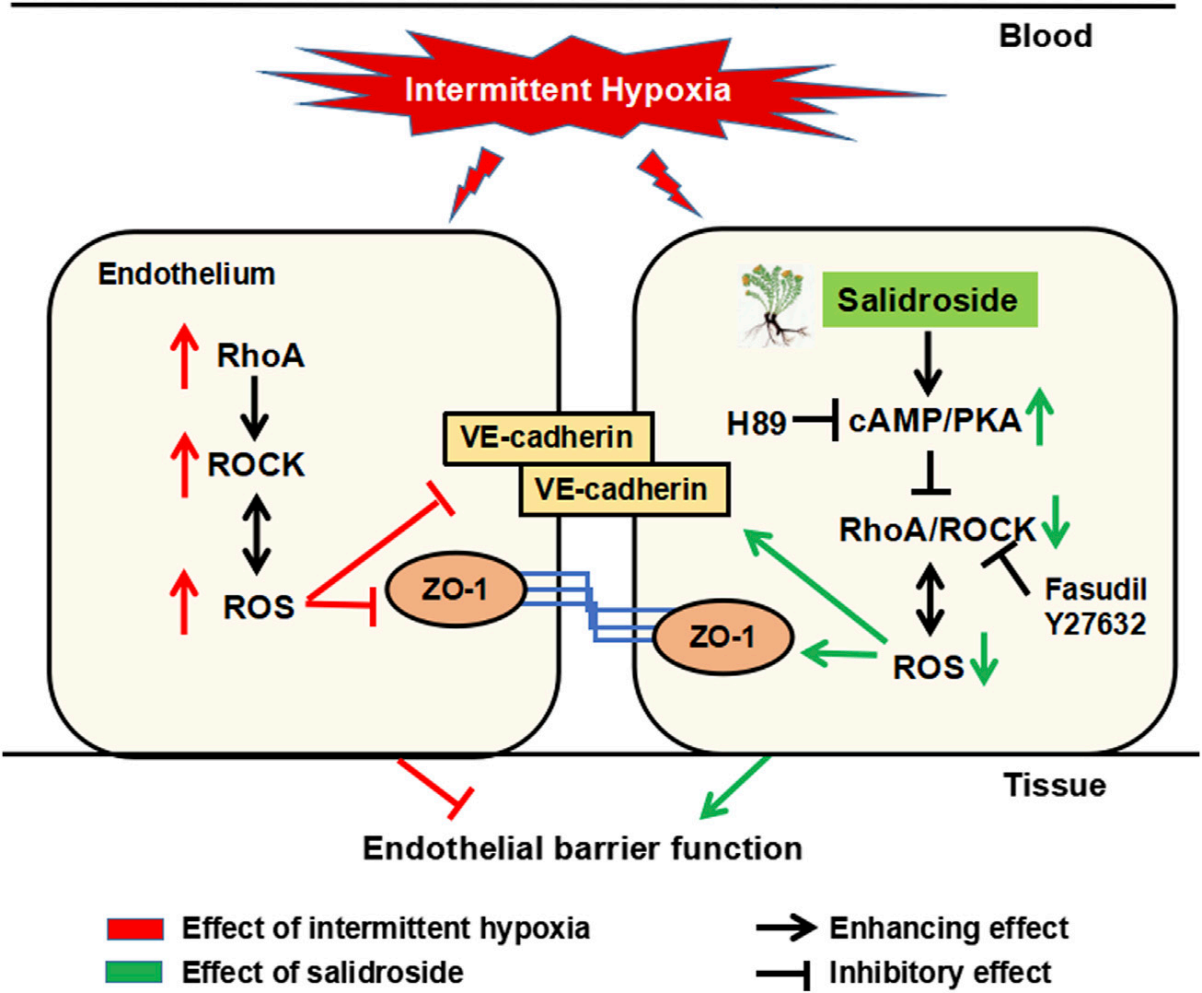
Schematic diagram of the potential mechanism by which salidroside protects against IH-induced ROS overproduction and barrier disruption in the endothelium. Salidroside increased intracellular cAMP levels, which subsequently activated PKA in HUVECs. Activated cAMP/PKA suppressed IH-induced elevation of RhoA and ROCK, and thereafter inhibited ROS generation. In turn, reduced ROS production inhibited RhoA/ROCK expression, breaking the vicious cycle of ROS augmentation induced by IH in the endothelium. IH, intermittent hypoxia; ROS, reactive oxygen species; RhoA, ras homolog gene family member A; ROCK, Rho-associated protein kinase; PKA, protein kinase A.

In summary, salidroside exerted significant barrier-protective and anti-atherosclerosis effects against IH *via* the cAMP/PKA/RhoA signaling pathway (Figure 7). We propose that this evidence supports potential therapeutic actions of salidroside in applications for OSA-associated vascular system protection.

## Data Availability

The original contributions presented in the study are included in the article/Supplementary Material, further inquiries can be directed to the corresponding author.
