# Supplementary material for: Salidroside Ameliorated Intermittent Hypoxia-Aggravated Endothelial Barrier Disruption and Atherosclerosis via the cAMP/PKA/RhoA Signaling Pathway
Source: Front Pharmacol. 2021 Aug 24;12:723922. doi: 10.3389/fphar.2021.723922 (PMC8421548; doi:10.3389/fphar.2021.723922)
Supplement: Supplementary file 1 [file DataSheet1.docx]

Supplementary Material

# Supplementary text

**1.1** **Biochemical analysis methods**

Blood fasting glucose, triglycerides and total cholesterol were measured using commercially available kits (BioSino, Beijing, China).

**1.2** **Oral glucose tolerance test and insulin tolerance test methods**

Blood was sampled via the tail vein from mice after 4 h of fasting. Blood fasting glucose was quantified using the glucose oxidase method at 0, 30, 60 and 120 min after glucose (2 g/kg) loading in the oral glucose tolerance test and at 40 and 90 min after subcutaneous injection of insulin (0.4 IU/kg) in the insulin tolerance test.

#
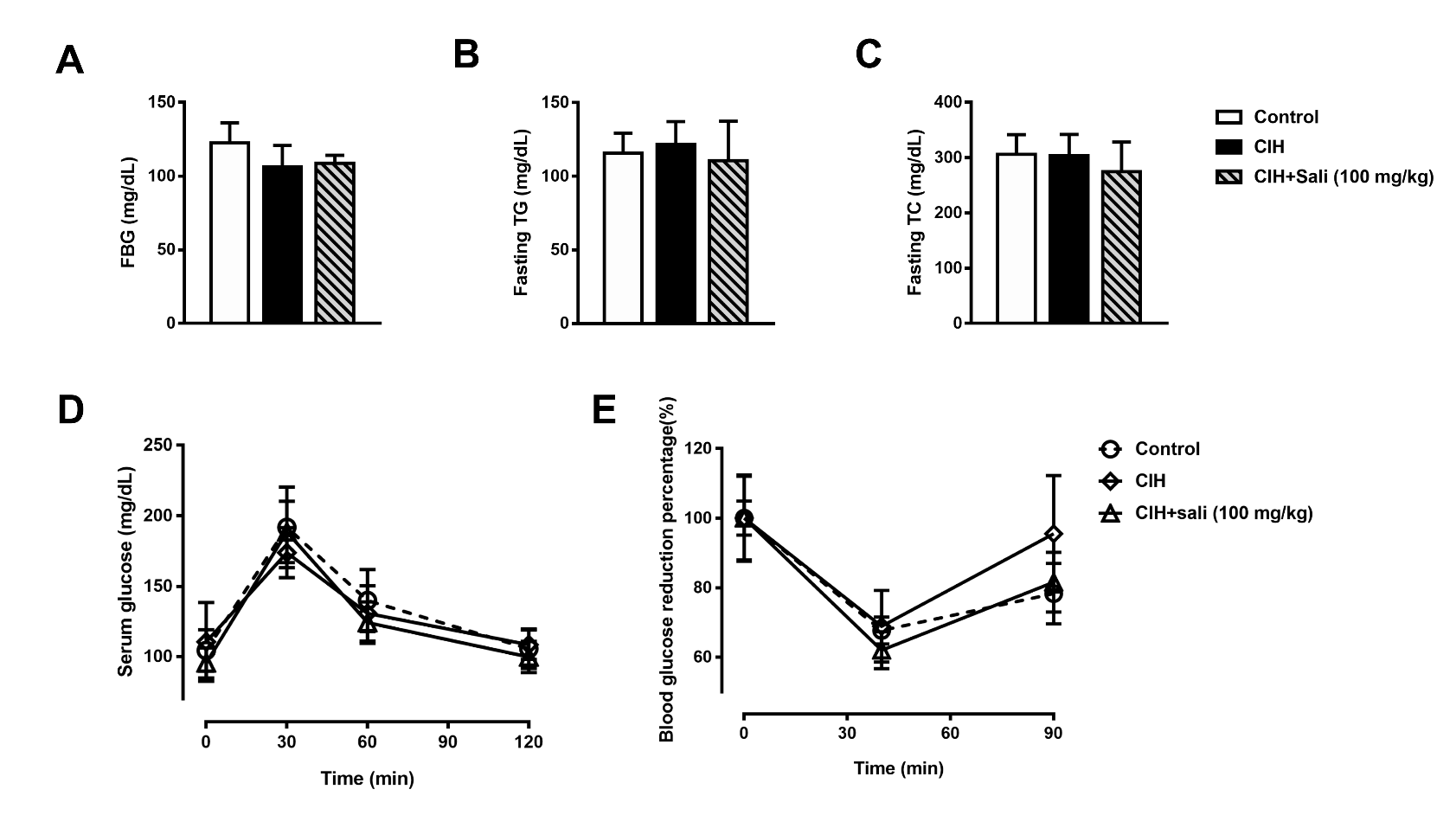
Supplementary Figure 1

**Supplementary Figure 1.** **Effects of salidroside on glucolipid metabolism and insulin sensitivity in CIH-exposed ApoE-/- mice.** (A) Blood fasting glucose, (B) TG, and (C) TC levels in CIH-exposed ApoE-/- mice treated with 100 mg/kg salidroside for 7 weeks (n = 8). (D) Blood glucose curve in oral glucose tolerance test on CIH-exposed ApoE-/- mice treated with 100 mg/kg salidroside for 7 weeks and orally administered 2 g/kg glucose after 4 h of fasting. (E) Blood glucose curve in insulin tolerance test on CIH-exposed ApoE-/- mice treated with 100 mg/kg salidroside for 7 weeks and injected with 0.4 IU /kg insulin after 4 h of fasting. CIH, chronic intermittent hypoxia; Sali, salidroside; TG, total triglycerides; TC, total cholesterol. All data are presented as mean ± SD. *p < 0.05 compared with CIH group.
